# Synthesis of Yellow-Fluorescent Carbon Nano-dots by Microplasma for Imaging and Photocatalytic Inactivation of Cancer Cells

**DOI:** 10.1186/s11671-021-03478-2

**Published:** 2021-01-21

**Authors:** Xing Qin, Jinlin Liu, Qing Zhang, Wantao Chen, Xiaoxia Zhong, Jie He

**Affiliations:** 1 Department of Oral and Maxillofacial-Head and Neck Surgery, Ninth People’s Hospital, Shanghai Jiao Tong University School of Medicine, 639 Zhizaoju Road, Shanghai, 200011 China; 2 Shanghai Key Laboratory of Stomatology and Shanghai Research Institute of Stomatology, National Clinical Research Center of Stomatology, Shanghai, 200011 People’s Republic of China; 3 Hunan Key Laboratory of Oral Health Research and Hunan 3D Printing Engineering Research Center of Oral Care and Hunan Clinical Research Center of Oral Major Diseases and Oral Health and Xiangya Stomatological Hospital and Xiangya School of Stomatology, Central South University, Changsha, 410008 Hunan People’s Republic of China; 4 State Key Laboratory of Advanced Optical Communication Systems and Networks, Key Laboratory for Laser Plasmas (Ministry of Education), School of Physics and Astronomy, Shanghai Jiao Tong University, Shanghai, 200240 People’s Republic of China

**Keywords:** Atmospheric pressure microplasma, Bioimaging, Carbon quantum dots, Photodynamic therapy, Yellow emission

## Abstract

In recent years, multifunctional nanoparticles with combined diagnostic and therapeutic functions show great promise in nanomedicine. In this study, we report the environmentally friendly synthesis of fluorescent carbon nano-dots such as carbon quantum dots (CQDs) by microplasma using *o*-phenylenediamine. The produced CQDs exhibited a wide absorption peaks at 380–500 nm and emitted bright yellow fluorescence with a peak at 550 nm. The CQDs were rapidly taken up by HeLa cancer cells. When excited under blue light, a bright yellow fluorescence signal and intense reactive oxygen species (ROS) were efficiently produced, enabling simultaneous fluorescent cancer cell imaging and photodynamic inactivation, with a 40% decrease in relative cell viability. Furthermore, about 98% cells were active after the incubation with 400 μg mL^−1^ CQDs in the dark, which revealed the excellent biocompatibility of CQDs. Hence, the newly prepared CQDs are thus demonstrated to be materials which might be effective and safe to use for in vivo bioimaging and imaging-guided cancer therapy.

## Introduction

Cancer remains a leading cause of death worldwide [1]. Multifunctional nanoparticles with both diagnostic and therapeutic functions have promising applications in nanomedicine. Simultaneous image-guided therapy is a new concept in cancer treatment and shows great promise with respect to the optimization of therapeutic efficiency. It can provide useful information regarding the size and location of tumors, the optimal time window for phototherapy, and therapeutic efficacy [2–4]. Photodynamic therapy (PDT) has been used to treat many kinds of cancers and other diseases owing to its spatiotemporal selectivity and non-invasive nature [5, 6]. Ideal photosensitizers generally possess the following characteristics: (1) highly efficient generation of reactive oxygen species (ROS), (2) good biocompatibility, and (3) water solubility [7]. However, current applications of PDT are limited by the poor water solubility, instability, and sub-optimal excitation wavelengths of photosensitizers. Therefore, the generation of photosensitizer substitutes with good water solubility and biocompatibility by environmentally friendly and low-cost methods is needed.

Carbon quantum dots (CQDs) have received tremendous attention due to their unique beneficial properties, such as simple and environmentally friendly synthesis, low toxicity, remarkable biocompatibility, excellent water solubility, and light stability [8]. CQDs have potential uses in cellular imaging, biosensing, targeted drug delivery, and other biomedical applications [9–13]. There are two major approaches for the synthesis of carbon dots, bottom-up and top-down approaches. Top-down methods include electrochemical oxidation, laser ablation, chemical oxidation, and ultrasonic synthesis methods. Bottom-up methods consist of hydrothermal treatment, microwave synthesis, and thermal decomposition [14–17]. However, the high temperature, high pressure, and strong acids required always lead to substantial energy consumption, complicated processes, and unavoidable harm to the environment. Therefore, novel environment-friendly synthetic methods emerge as the times require. As it has been reported, CQDs can be produced within just a few minutes using a microplasma-liquid method without high temperature condition, large energy input, and laborious procedures [18–20]. Microplasmas supply a unique physicochemical environment to both fundamental studies and applications involving advanced materials. The chemical and electronic environments provided by the microplasmas are highly nonequilibrium and can store energy. In this environment, a large number of electrons, ions, free radicals, and other excited-ionized-active substances can be produced [21, 22]. Although *o*-phenylenediamine is a raw material for carbon nano-dots synthesis, it has not been used in microplasma synthesis [23–25].

In this study, *o*-phenylenediamine was used as a raw material to synthesize CQDs by microplasma treatment. The CQDs generated by this method were uniform in size (around 3.2 nm in diameter) and exhibited an emission peak at around 550 nm. We demonstrated that the newly synthesized CQDs could produce a large amount of ROS under light conditions. In vitro, CQDs could be absorbed by HeLa tumor cells and emitted yellow light under blue wavelength excitation at 420–500 nm with low toxicity. We also observed the inactivation of HeLa tumor cells under irradiation at 460 nm. These results suggest that the newly prepared CQDs might be promising materials for in vivo bioimaging, imaging-guided or targeted cancer therapy.

## Results and Discussion

### Characterization of CQDs

**Fig. 1:**
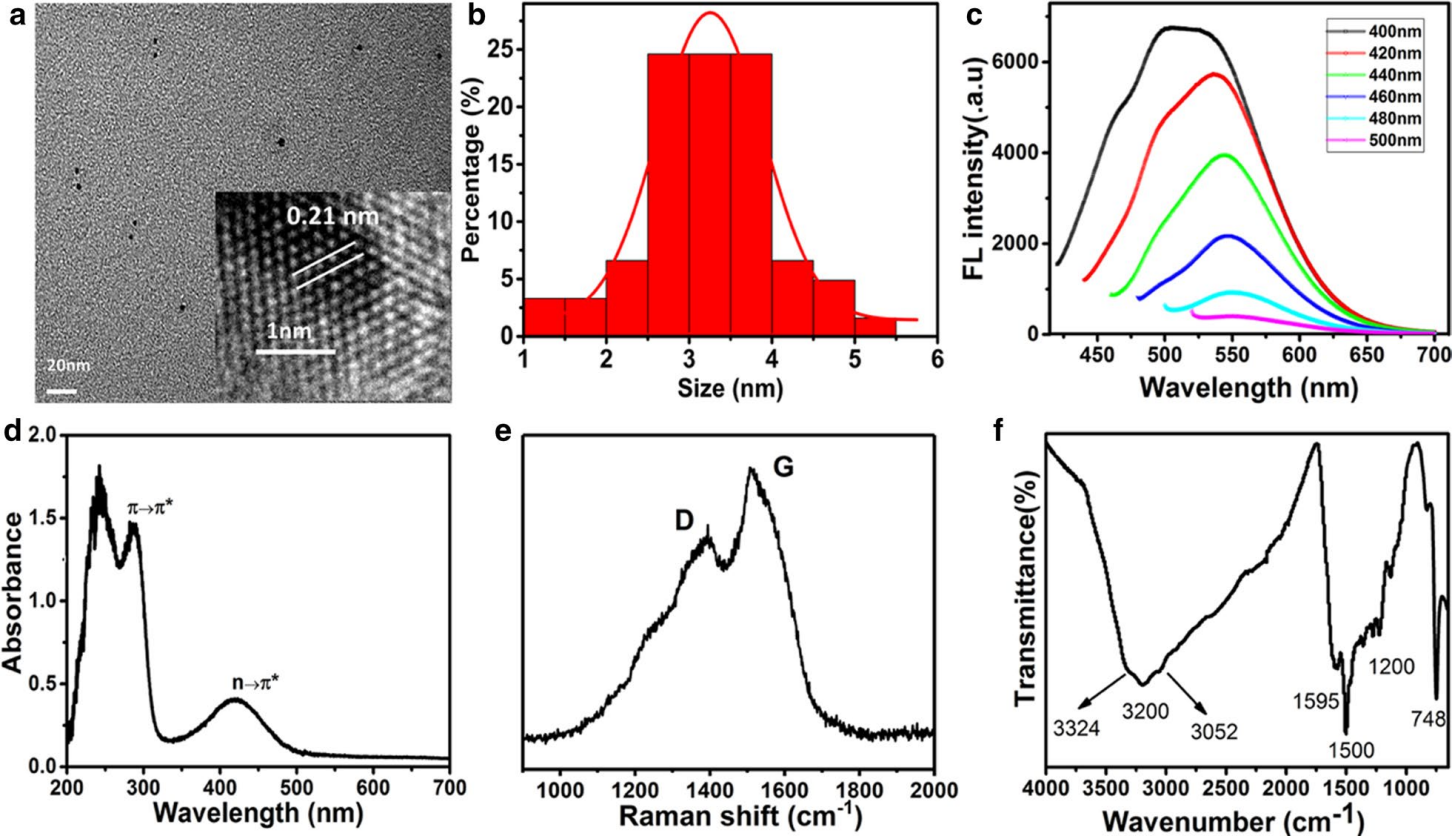
Characterizations of CQDs. **A** TEM images of CQDs (inset, high resolution TEM images); **B** Size distributions of CQDs; **C** UV–vis absorption spectra of the CQDs; **D** The FL spectrum of the CQDs with excitation wavelengths from 400 to 500 nm in 20 nm increments; **E**, **F** Raman spectrum of CQDs and FTIR spectrum of CQDs

The yellow-emissive CQDs in this study are prepared in a facile and environmentally friendly manner by a microplasma method using *o*-phenylenediamine as the carbon precursor. Although the method of microplasma processing has been reported to be used for carbon nano-dots synthesis, there exist rare relative researches. Figure 1A shows transmission electron microscope (TEM) images of the CQD particles. The particles produced by microplasma were cyclo-sharp or oval-shaped with an average diameter of 3.2 nm. As shown in the high-resolution image Fig. 1A (inset), the lattice distance in the CQDs is 0.21 nm, belonging to the (1,1,0) plane of graphite. The Raman spectrum shows that D mode, named as disorder induced mode, located around 1342 cm^−1^ and G mode centers around 1507 cm^−1^, respectively, due to the result of *sp*^3^ and *sp*^2^-hybridization of carbon (Fig. 1E). It is known that the intensity of D mode compared with G mode depends on the size of the graphite micro-crystals in the sample. The higher disorder of the sample leads to the higher intensity ratio of ID/IG and the smaller graphite micro-crystal. Furthermore, the D and G mode of CQD powders can also be viewed as small graphite flakes with a relatively high intensity ratio of ID/IG (0.77).

**Fig. 2:**
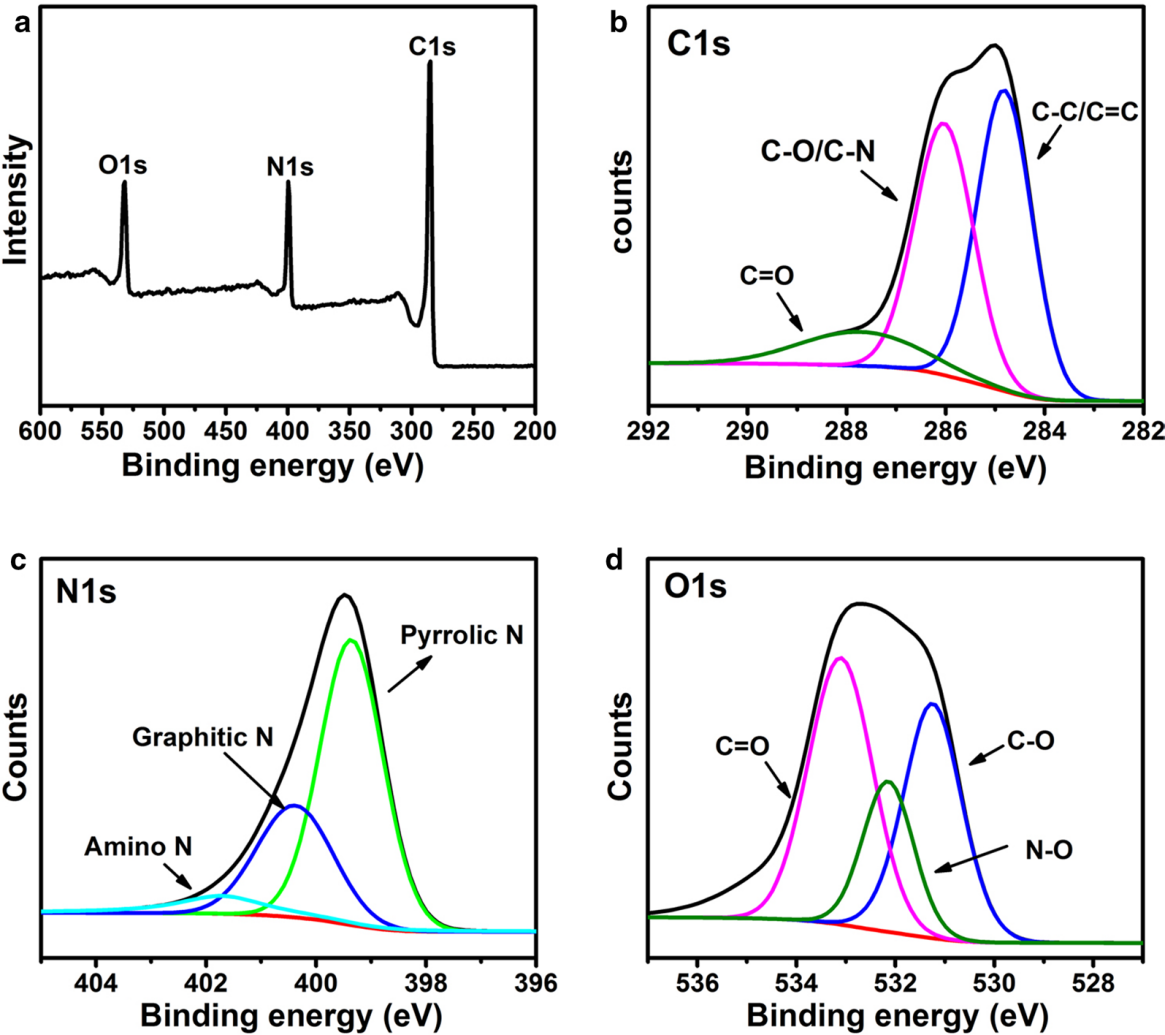
XPS spectra of CQDs. **A** Full-scale XPS spectra of the CQDs; **B** High-resolution of C 1*s* spectrum; **C** high-resolution of N 1*s* spectrum; **D** High-resolution of O 1*s* spectrum

FTIR and XPS are powerful tools to characterize the chemical composition and structure of carbon-based materials. FTIR data for CQDs were recorded in the range of 400–4000 cm^−1^, as shown in Fig. 1F. The FTIR spectrum revealed that the CQDs mainly contain amine (3052 and 3324 cm^−1^), OH (3200 cm^−1^), C=O (1595 cm^−1^), C–N/C–O (1200 cm^−1^), C=C (1500 cm^−1^) and CH (748 cm^−1^) functional groups or chemical bonds [26, 27]. The surface components of the CQDs, as determined by XPS, were consistent with the FTIR results. The full spectrum presented in Fig. 2A showed three typical peaks: C 1*s* (285 eV), N 1*s* (400 eV), and O 1*s* (531 eV).

As shown in Fig. 2B–D, a C 1*s* analysis revealed the presence of *sp*^2^/*sp*^3^ carbons (C–C/C=C, 284.8 eV), nitrous carbons (C–O/C–N, 285.9 eV), and carbonyl carbons (C=O, 287.7 eV). The N 1*s* band was deconvoluted into three peaks at 399.3, 400.3, and 401.7 eV, which correspond to pyrrolic N, graphitic N, and amino N, respectively. The O 1*s* band contained peaks at 531.6 and 533.1 eV for C–O and C=O, respectively [28, 29]. Importantly, the existence of these above functional groups endowed CQDs with favorable solubility. Furthermore, the optical properties of CQDs were investigated using fluorescence spectroscopy and UV–Vis absorption. The fluorescence emission spectra of CQDs are shown in Fig. 1C. The prepared CQDs exhibit an excitation-dependent fluorescence emission behavior. When excited at wavelengths from 400 to 500 nm, the maximum fluorescence emission peak red-shifted from 473 to 519 nm and the fluorescence intensity decreased sharply [30]. As shown in Fig. 1D, the UV–vis spectra of CQD featured a strong absorption peak in the wavelength range of 400–490 nm. The CQDs exhibited two characteristic absorption peaks at 280 and 420 nm, which referred to π–π* (aromatic C=C) and n–π* (carboxyl and/or C–N) transitions, respectively [31, 32]. Therefore, these optical properties of CQDs provided feasibility for simultaneous biological imaging and photodynamic inactivation.

### Bioimaging and cytotoxicity of CQDs

**Fig. 3:**
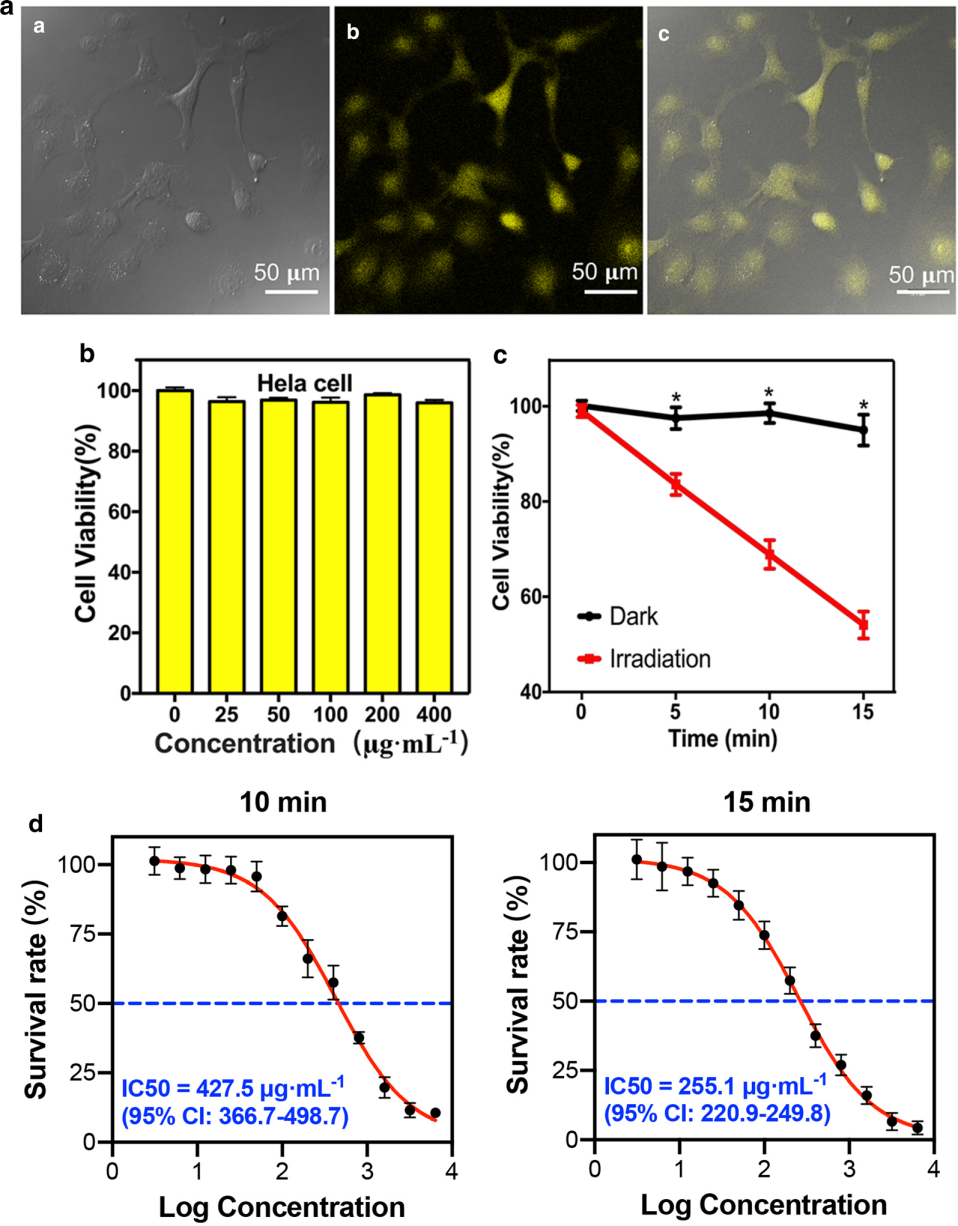
Application of CQDs. **A** CLSM imaging of HeLa cells labeled with CQDs; **B** In vitro cytotoxicity test of CQDs; **C** Relative viability of HeLa cells incubated with control solution or CQDs (200 μg mL^−1^) and exposed to blue light (460 nm, 30 mW cm^−2^) for 5 min, 10 min, and 15 min; **D** The IC50 of excited CQDs on Hela cells after exposure to blue light for 10 and 15 min (*P < 0.05)

To assess the capability of CQDs for bioimaging and cell labeling, in vitro cellular imaging using CQDs was investigated on HeLa cells by a confocal laser scanning microscope (CLSM). Hela cells were incubated with 200 μg mL^−1^ CQDs for 6 h and then prepared for CLSM detection. As a result, HeLa cells showed bright yellow fluorescence evenly distributed throughout the cell (Fig. 3A). More importantly, it should be noted that low CQDs concentration of 200 μg mL^−1^ was enough to label Hela cells with yellow fluorescence, which further specified the possibility of CQDs in cell imaging. As it has been reported, carbon nanoparticles always exhibited strong emission only in the blue-light region, while the long-wavelength emissions were usually weak. Under UV excitation, biological tissues frequently showed blue autofluorescence and were vulnerable to receive photodamage, which seriously hinders the biological imaging analysis applications of carbon nanoparticles with short-wavelength emissions [33]. Therefore, the development of carbon nanoparticles with long-wavelength emissions was widely concerned. In the present study, the as-prepared CQDs showed bright yellow fluorescence under the excitation of a 400–450 nm light. The excited yellow fluorescence thereby might enable CQDs to be used for detection of deep-seated tumors. However, there is still a long way to go before practical applications in human cancer imaging.

Besides luminescence characteristic, high biocompatibility, and low toxicity are always required if CQDs are intended to be developed as a potential bio-labeling reagent. For biomedical applications, materials must be highly biocompatible at recommended dosages. To examine cytotoxicity, HeLa cells were treated with CQDs at final concentrations ranging from 0 to 400 μg mL^−1^ for 24 h. As shown in Fig. 3B, more than 95% of cells survived as determined by MTT (3-(4,5-dimethylthiazolyl-2-yl)-2,5-diphenyltetrazolium bromide) assays, which revealed that CQDs were practically non-toxic.

These data suggest that the newly generated CQDs with excited yellow fluorescence have low cytotoxicity and high biocompatibility, which facilitate their promising prospect of biological imaging.

### Efficacy of Photodynamic Therapy

#### Cancer Cell Inactivation

As shown in Fig. 3C, viability did not differ between HeLa cells treated with CQDs or blue light alone. Simultaneous treatment with CQDs and blue light markedly reduced the cell viability of Hela cells, depending on the duration of photo-exposure. After irradiation at 460 nm for 15 min, the CQDs displayed remarkable antitumor activity; the viability of HeLa cells decreased by about 40% at a concentration of 200 μg mL^−1^. To further detect the effects of the excited CQDs, MTT assays were implemented to evaluate the half maximal inhibitory concentration (IC50) of excited CQDs to Hela cells. As a result, the IC50 of CQDs after being excited on Hela cells was about 427.5 μg mL^−1^ (95% CI 366.7–498.7 μg mL^−1^) after a 10-min irradiation and was approximately 255.1 μg mL^−1^ (95% CI 220.9–249.8 μg mL^−1^) after exposure to blue light for 15 min.

These results indicated that the excited CQDs could effectively kill tumor cells like some clinical anticancer drugs, such as Photofrin [34], which prompted the promising value of CQDs in imaging-guided antitumor therapy.

### ROS Generation of CQDs

**Fig. 4:**
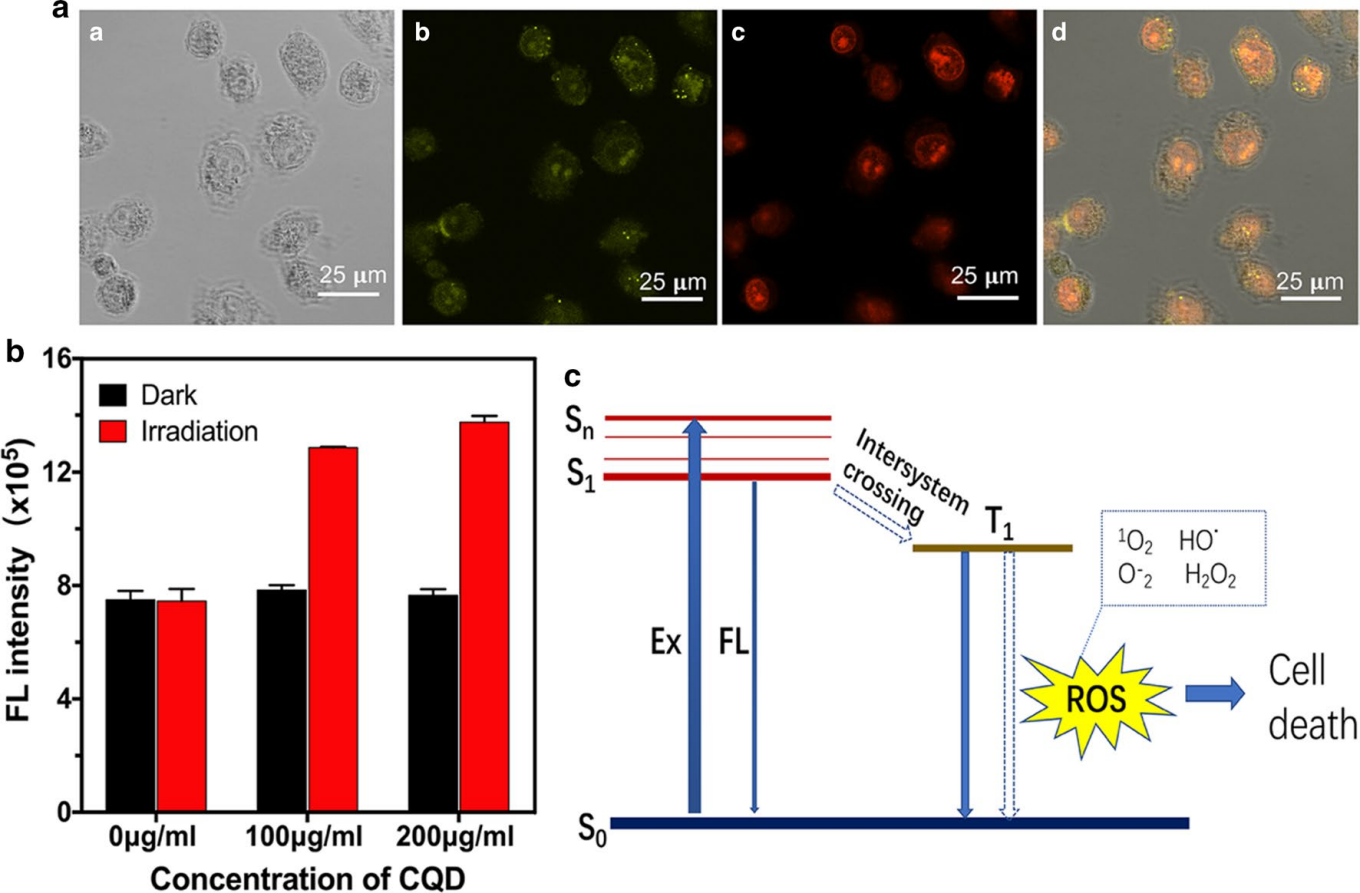
Intracellular ROS generation. **A** Fluorescence images of HeLa, (a) Bright field transmission image, (b) CQDs fluorescence image collected in the range of 400–450 nm, (c) ROS detection reagent fluorescence image captured in the range of 510–530 nm, and (d) the merged image; **B** The intracellular ROS production for various concentrations of CQDs with or without irradiation for 15 min; **C** a simplified energy level diagram shows potential fluorescence and cell death energy transfer pathways. (S0, ground state of the fluorophore molecule; S1, first singlet excited state; Sn (*n* > 1); T1, first triplet excited state; Ex, excitation by photon absorption; FL, fluorescence)

During PDT, cancer cells can be killed by cytotoxic ROS generated by the endocytosed photosensitizer under appropriate irradiation conditions [35, 36]. ROS can inactivate target cells by apoptosis or necrosis with little side effects via PDT in several diseases [37–40]. Inspection of Fig. 4A showed that ROS reagent emits red fluorescence, indicating the generation of ROS. Moreover, the red signal of ROS was well overlapped with the fluorescence of CQDs, which means that the generation of ROS was closely associated with the uptake of CQDs by tumor cells. As shown in Fig. 4B, compared with the control and no laser irradiation groups, the experimental groups under 460 nm laser irradiation for 15 min showed obvious ROS generation. Our results indicated that CQDs could significantly promote the production of the intracellular ROS under the irradiation of 460 nm laser and had great potential for application in PDT. In principle, the CQDs can be excited from the ground state (S0 in Fig. 4C) to an excited state (Sn in Fig. 4C), and the efficiency of this process is determined by the intensity of the light source and the extinction coefficient. After solvent-mediated relaxation, CQDs remain at the lowest vibration level of the first singlet excited state. Due to the rapid vibrational relaxation following excitation, the energy of the photon emitted from the first singlet excited state (S1 in Fig. 4C) is lower than the energy of the excitation photon, resulting in a wavelength increase. Fluorescence imaging utilizes the CQDs transition from S0 to Sn to S1 [41]. The CQDs were ingested by HeLa tumor cells and emitted fluorescence when illuminated by a suitable wavelength source, thereby allowing the cells to be labeled. S1 can return to the S0 state by fluorescence or by intersystem crossing to a non-fluorescent triplet excited state (T1 in Fig. 4C) [7, 42]. The fluorescent group of T1 is especially active in electron transfer reactions, generating superoxide free radicals and subsequently resulting in fluorescent group degradation. The energy from T1 transferred to molecular oxygen would produce an excited singlet oxygen oxidizing agent that is stronger than ground state molecular oxygen. The superoxide radicals and singlet oxygen, as well as other ROS, including OH and H_2_O_2_, react with nearby biological molecules to exert phototoxicity, leading to cell death. After the CQDs were taken up by HeLa cells, illumination resulted in the transfer of the singlet state to the triplet state through the intersystem, and the process of energy transfer produced ROS and ultimately led to cell death. Under the appropriate wavelength source, the CQDs undergo two types of energy transfer. Fluorescence was emitted to mark HeLa tumor cells, and HeLa cells were killed by ROS. Our experimental data revealed the good biocompatibility of the newly produced CQDs in the dark and the tumor-killing efficiency under light conditions. Hence, the CQDs could be used as a photosensitizer for tumor cells and tissues.

In addition, it is still an unsolved problem how CQDs can target tumor accurately and kill tumor cells more effectively. The existing abundance of surface functional hydrophilic groups (carboxyl, carbonyl, epoxy, hydroxyl, hydroxyl, etc.) enables carbon dots to conjugate with specific antibody which could precisely target tumors, and this requires tumor have specific biomarkers. Simultaneously, carbon dots could also serve as a tool for drug and gene delivery owing to their high surface area to volume ratio [43]. Considering the excellent biocompatibility, small size for internalization by tumor cells, being rich in surface functional moieties and the bioimaging potentials, carbon dots (include CQDs) are believed to become promising theranostic candidates for tumor therapy. However, there still exist challenges and many unresolved problems for the applications of carbon dots in nanomedicine and bioimaging. More efforts should be taken to promote the translation of carbon dot-related nanomedicine from bench to bedside in the future.

## Conclusions

In summary, we synthesized photoluminescent CQDs using *o*-phenylenediamine by the plasma method. Excited by a blue laser, CQDs with a diameter of about 3.2 nm emitted yellow fluorescence. Raman, UV–vis, FTIR, and XPS results showed that more carbon atoms were involved in *sp*^2^ hybridization, forming a new organic group. CQDs synthesized by the plasma method proved to be an effective probe in cell imaging experiments, and the yellow fluorescence emitted by CQDs can clearly mark HeLa cells. Moreover, the synthesized CQDs showed favorable solubility, non-toxic, and high biocompatibility, which could accelerate their capability of bio-imaging. In addition, the excited CQDs could effectively kill Hela cells by ROS generation, which clearly exhibited satisfactory photodynamic cytotoxicity of CQDs in vitro and supported their applications in PDT.

## Experimental Methods

### Synthesis of Carbon Quantum Dots

The microplasma processing system is summarized in Additional file 1: Figure S1. A hollow stainless pipe with an inner diameter of 180 μm was connected to a high-voltage direct current power source (Tianjin Dongwen High-Voltage Supply Co., Ltd., Tianjin, China) and was kept at 2 mm above the surface of the solution. A Pt electrode (DJS-1; Shanghai INESA Scientific Instrument Co., Ltd., Shanghai, China) was connected to the cathode of the power source and immersed in the solution. Then, 400 mg of *o*-phenylenediamine (Shanghai, China) was dissolved in 40 mL of deionized water, and 20 mL of the *o*-phenylenediamine solution was added to a Petri dish and stirred using a magnetic stirrer. During microplasma treatment, argon (Ar) gas flowed through the pipe at a flow rate of 60 sccm, and the DC current was kept at 17 mA. After 10 min of plasma treatment, the brownish-black product was dialyzed using a dialysis membrane (molecular weight cutoff, 500 Da) against 2 L of deionized water for 12 h and subsequently filtered through a 0.22 μm ultrafiltration membrane. Finally, pure CQDs were obtained by freeze drying.

### Characterization of the Structure, Composition, and Optical Properties of CQDs

The size and morphology of the CQDs were characterized by TEM using a JEM-2100F system (JEOL, Tokyo, Japan). Fluorescence spectroscopy was performed using a Perkin Elmer LS 55 Luminescence Spectrometer (Waltham, MA, USA). The UV/Vis absorption spectra were measured using the Varian Cary 50 UV–VIS spectrophotometer (Palo Alto, CA, USA). FTIR spectra were obtained using a Nicolet 6700 spectroscope (Thermo Scientific, Waltham, MA, USA), and Raman spectroscopy was performed using the 800 UV micro-Raman spectrometer (Invia-reflex, UK). XPS experiments were performed using an Axis Ultra DLD system (Shimadzu/Kratos Analytical Ltd., Kyoto, Japan).

### Cell Culture and Cytotoxicity Assay

HeLa cells (ATCC, Manassas, VA, USA) were cultured in Dulbecco's Modified Eagle Medium (DMEM) containing 10% FBS and 1% penicillin–streptomycin at 37 °C in a humidified 5% CO_2_ atmosphere. For cytotoxicity studies of CQDs, cells were counted and seeded in 96-well plates containing 200 μL of complete medium at a density of 6000 cells per well. After 24 h of culture, cells were incubated with CQDs at concentrations of 0, 25, 50, 100, 200, and 400 μg mL^−1^ for another 24 h, and then, the cell viabilities were detected using MTT assays to assess the cytotoxicity of CQDs. Briefly, these solutions were replaced with 100 μL MTT test solution (0.5 mg mL^−1^) and incubated for 4 h in an incubator in the dark. The supernatant was removed and the crystals were dissolved in dimethyl sulfoxide (DMSO). Finally, the absorbance of each well was measured at 490 nm. The optical density was related to cell viability by assuming 100% viability for the control sample without CQDs.

MTT assay was also used to assess the IC50 of excited CQDs on Hela cells. Briefly, Hela cells in 96-well plate were incubated with CQDs at concentrations of 0, 6.25, 12.5, 25, 50, 100, 200, 400, 800, 1600, 3200, and 6400 μg mL^−1^ for 24 h in the incubator, treated with light at 460 nm for 10 min or 15 min separately, and then cultured for another 24 h. The cell viability of each well was detected using MTT assay and the data was used for IC50 evaluation.

### Cell Imaging

Cells at a concentration of 2 × 10^4^ mL^−1^ were seeded in a confocal dish (diameter = 15 mm), cultured for 24 h, and washed with PBS twice to ensure no dead cells. A CQD solution (200 μg mL^−1^; pH 7) was added and the cells were incubated for 6 h. The cells were subsequently washed with PBS three times to remove unbound CQDs and fixed with 4% paraformaldehyde. Then, the samples were observed using a CLSM (LSM510, Zeiss, Germany) with excitement at wavelengths ranging from 400 to 450 nm.

### Photodynamic Therapy and ROS Measurement

To investigate antitumor effects, HeLa cells were incubated with 200 μg mL^−1^ CQDs for 24 h at 37 °C in the dark and treated with light at 460 nm (30 mW cm^−2^) for 5, 10, and 15 min. After 24 h of incubation, a standard MTT assay was performed to determine the relative cell viability. The intracellular generation of ROS was detected chemically using the spectrophotometric method with Fluorometric Intracellular Ros Assay Kit (sigma, USA). Cells were cultured in a confocal dish (diameter = 15 mm) overnight for cell attachment. Then, the cells were incubated with 200 μg mL^−1^ CQDs for 4 h. Subsequently, 100 μL/well of Master Reaction Mix was added. After incubation for 1 h, the cells were fixed with 4% paraformaldehyde for 10 min, and fluorescence images of the cells were observed by CLSM. As for ROS production detection, cells were cultured in 96-well plates with 200 μL culture medium. After 24 h incubation, the medium was replaced with 100 μL CQDs solution at the concentrations of 0, 100, 200 μg mL^−1^, and the cells were incubated for another 4 h. Subsequently, the samples were washed three times with PBS, irradiated for 15 min or not, and incubated with 100 μL/well Master Reaction Mix for 1 h. Finally, the fluorescence intensity was detected using a fluorescence reader (520 nm excitation, 605 nm emission).

### Statistical Analyses

Experiments involved in this study were repeated for three times, and statistical analyses were performed using SPSS19.0. The differences between the two groups were compared using Mann–Whitney *U* test. The IC50 of excited CQDs on Hela cells was evaluated by using nonlinear regression. *P* < 0.05 was considered statistically significant.

## Supplementary Information

**Additional file 1: Figure S1.** A summary of the micro plasma processing system.

## Data Availability

All data and materials of the current study are available from the corresponding author on reasonable request.

## Abbreviations

PDT: Photodynamic therapy
ROS: Reactive oxygen species
CQDs: Carbon quantum dots
TEM: Transmission electron microscope
CLSM: Confocal laser scanning microscope
DMEM: Dulbecco's modified eagle medium
MTT: 3-(4,5-Dimethylthiazolyl-2-yl)-2,5-diphenyltetrazolium bromide
DMSO: Dimethyl sulfoxide
IC50: Half maximal inhibitory concentration

## References

[1] Bray F, Ferlay J, Soerjomataram I, Siegel RL, Torre LA, Jemal A (2018). Global cancer statistics 2018: GLOBOCAN estimates of incidence and mortality worldwide for 36 cancers in 185 countries. CA Cancer J Clin.

[2] Shen J, Zhao L, Han G (2013). Lanthanide-doped upconverting luminescent nanoparticle platforms for optical imaging-guided drug delivery and therapy. Adv Drug Deliv Rev.

[3] de Jong M, Essers J, van Weerden WM (2014). Imaging preclinical tumour models: improving translational power. Nat Rev Cancer.

[4] Lammers T, Aime S, Hennink WE, Storm G, Kiessling F (2011). Theranostic nanomedicine. Acc Chem Res.

[5] Oniszczuk A, Wojtunik-Kulesza KA, Oniszczuk T, Kasprzak K (2016). The potential of photodynamic therapy (PDT)-experimental investigations and clinical use. Biomed Pharmacother.

[6] Monro S, Colon KL, Yin H, Roque J, Konda P, Gujar S (2019). Transition metal complexes and photodynamic therapy from a tumor-centered approach: challenges, opportunities, and highlights from the development of TLD1433. Chem Rev.

[7] Lovell JF, Liu TW, Chen J, Zheng G (2010). Activatable photosensitizers for imaging and therapy. Chem Rev.

[8] Ghosal K, Ghosh A (2019). Carbon dots: the next generation platform for biomedical applications. Mater Sci Eng C Mater Biol Appl.

[9] Mishra V, Patil A, Thakur S, Kesharwani P (2018). Carbon dots: emerging theranostic nanoarchitectures. Drug Discov Today.

[10] Liu W, Li C, Ren Y, Sun X, Pan W, Li Y (2016). Carbon dots: surface engineering and applications. J Mater Chem B.

[11] Zhang L, Wang D, Huang H, Liu L, Zhou Y, Xia X (2016). Preparation of gold-carbon dots and ratiometric fluorescence cellular imaging. ACS Appl Mater Interfaces.

[12] Yuan Y, Guo B, Hao L, Liu N, Lin Y, Guo W (2017). Doxorubicin-loaded environmentally friendly carbon dots as a novel drug delivery system for nucleus targeted cancer therapy. Colloids Surf B Biointerfaces.

[13] Du J, Xu N, Fan J, Sun W, Peng X (2019). Carbon dots for in vivo bioimaging and theranostics. Small.

[14] Sun YP, Zhou B, Lin Y, Wang W, Fernando KA, Pathak P (2006). Quantum-sized carbon dots for bright and colorful photoluminescence. J Am Chem Soc.

[15] Dong X, Shi Y, Huang W, Chen P, Li LJ (2010). Electrical detection of DNA hybridization with single-base specificity using transistors based on CVD-grown graphene sheets. Adv Mater.

[16] Xu X, Ray R, Gu Y, Ploehn HJ, Gearheart L, Raker K (2004). Electrophoretic analysis and purification of fluorescent single-walled carbon nanotube fragments. J Am Chem Soc.

[17] Du F, Ming Y, Zeng F, Yu C, Wu S (2013). A low cytotoxic and ratiometric fluorescent nanosensor based on carbon-dots for intracellular pH sensing and mapping. Nanotechnology.

[18] Wang Q, Zhang Q, Chen Y, He J, Jiang K, Hu Z (2018). Blue luminescent amorphous carbon nanoparticles synthesized by microplasma processing of folic acid. Plasma Process Polym.

[19] Huang X, Li Y, Zhong X, Rider AE, Ostrikov KK (2015). Fast microplasma synthesis of blue luminescent carbon quantum dots at ambient conditions. Plasma Process Polym.

[20] Lin L, Wang Q (2015). Microplasma: a new generation of technology for functional nanomaterial synthesis. Plasma Chem Plasma Process.

[21] Chiang WH, Mariotti D, Sankaran RM, Eden JG, Ostrikov K (2020). Microplasmas for advanced materials and devices. Adv Mater.

[22] Mariotti D, Patel J, Švrček V, Maguire P (2012). Plasma-liquid interactions at atmospheric pressure for nanomaterials synthesis and surface engineering. Plasma Process Polym.

[23] Zhang S, Ji X, Liu J, Wang Q, Jin L (2020). One-step synthesis of yellow-emissive carbon dots with a large Stokes shift and their application in fluorimetric imaging of intracellular pH. Spectrochim Acta A Mol Biomol Spectrosc.

[24] Tian M, Liu Y, Wang Y, Zhang Y (2019). Facile synthesis of yellow fluorescent carbon dots for highly sensitive sensing of cobalt ions and biological imaging. Anal Methods UK.

[25] Tian M, Liu Y, Wang Y, Zhang Y (2019). Yellow-emitting carbon dots for selective detecting 4-NP in aqueous media and living biological imaging. Spectrochim Acta A Mol Biomol Spectrosc.

[26] Fang HY, Huang WM, Chen DH (2019). One-step synthesis of positively charged bifunctional carbon dot/silver composite nanoparticles for killing and fluorescence imaging of Gram-negative bacteria. Nanotechnology.

[27] Zhang Y, Ishikawa K, Mozetič M, Tsutsumi T, Kondo H, Sekine M (2019). Polyethylene terephthalate (PET) surface modification by VUV and neutral active species in remote oxygen or hydrogen plasmas. Plasma Process Polym.

[28] Lu S, Sui L, Liu J, Zhu S, Chen A, Jin M (2017). Near-infrared photoluminescent polymer-carbon nanodots with two-photon fluorescence. Adv Mater.

[29] Jiang K, Sun S, Zhang L, Wang Y, Cai C, Lin H (2015). Bright-yellow-emissive N-doped carbon dots: preparation, cellular imaging, and bifunctional sensing. ACS Appl Mater Interfaces.

[30] Tang L, Ji R, Cao X, Lin J, Jiang H, Li X, Teng KS, Luk CM, Zeng S, Hao J, Lau SP (2012). Deep ultraviolet photoluminescence of water-soluble self-passivated graphene quantum dots. ACS Nano.

[31] Wang L, Zhou HS (2014). Green synthesis of luminescent nitrogen-doped carbon dots from milk and its imaging application. Anal Chem.

[32] Xu M, He G, Li Z, He F, Gao F, Su Y (2014). A green heterogeneous synthesis of N-doped carbon dots and their photoluminescence applications in solid and aqueous states. Nanoscale.

[33] Ko HY, Chang YW, Paramasivam G, Jeong MS, Cho S, Kim S (2013). In vivo imaging of tumour bearing near-infrared fluorescence-emitting carbon nanodots derived from tire soot. Chem Commun (Camb).

[34] Beack S, Kong WH, Jung HS, Do IH, Han S, Kim H (2015). Photodynamic therapy of melanoma skin cancer using carbon dot—chlorin e6–hyaluronate conjugate. Acta Biomater.

[35] Jin Y, Li Y, Ma X, Zha Z, Shi L, Tian J (2014). Encapsulating tantalum oxide into polypyrrole nanoparticles for X-ray CT/photoacoustic bimodal imaging-guided photothermal ablation of cancer. Biomaterials.

[36] Li Y, Liu Z, Hou Y, Yang G, Fei X, Zhao H (2017). Multifunctional nanoplatform based on black phosphorus quantum dots for bioimaging and photodynamic/photothermal synergistic cancer therapy. ACS Appl Mater Interfaces.

[37] Rkein AM, Ozog DM (2014). Photodynamic therapy. Dermatol Clin.

[38] Aniogo EC, Plackal Adimuriyil George B, Abrahamse H (2019). The role of photodynamic therapy on multidrug resistant breast cancer. Cancer Cell Int.

[39] Hwang HS, Shin H, Han J, Na K (2018). Combination of photodynamic therapy (PDT) and anti-tumor immunity in cancer therapy. J Pharm Investig.

[40] Shi X, Zhang CY, Gao J, Wang Z (2019). Recent advances in photodynamic therapy for cancer and infectious diseases. Wiley Interdiscip Rev Nanomed Nanobiotechnol.

[41] Turro NJ, Ramamurthy V, Scaiano JC (2012). Modern molecular photochemistry of organic molecules. Photochem Photobiol.

[42] Zheng Q, Juette MF, Jockusch S, Wasserman MR, Zhou Z, Altman RB (2014). Ultra-stable organic fluorophores for single-molecule research. Chem Soc Rev.

[43] Ostadhossein F, Pan D (2017). Functional carbon nanodots for multiscale imaging and therapy. Wiley Interdiscip Rev Nanomed Nanobiotechnol.

