# Supplementary figures and images for: Synthesis of Yellow-Fluorescent Carbon Nano-dots by Microplasma for Imaging and Photocatalytic Inactivation of Cancer Cells
Source: Nanoscale Res Lett. 2021 Jan 21;16:14. doi: 10.1186/s11671-021-03478-2 (PMC7818297; doi:10.1186/s11671-021-03478-2)

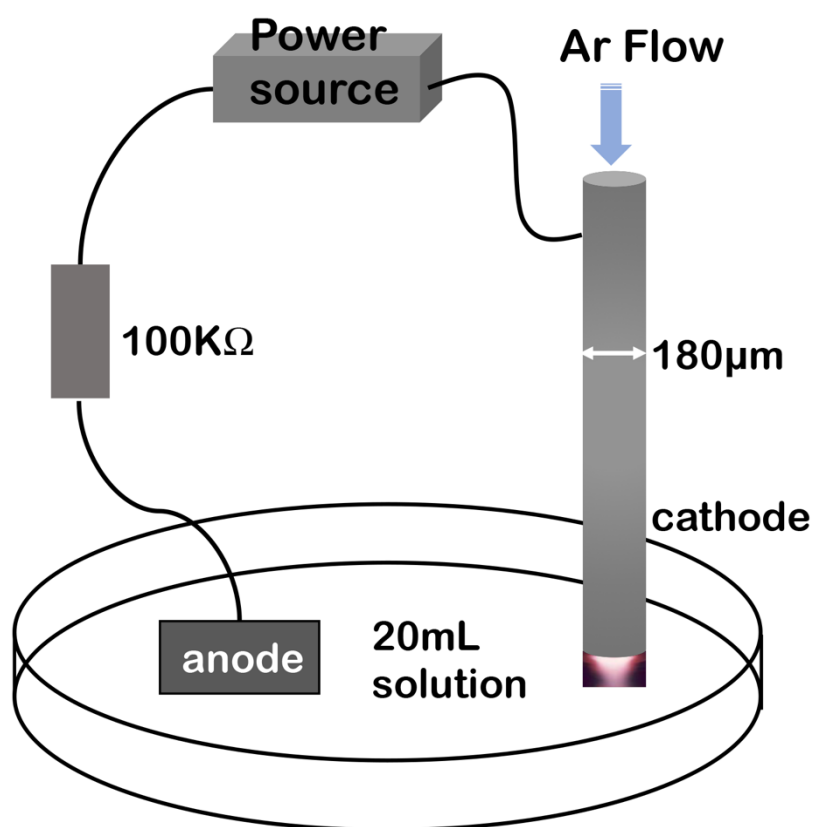

Supplement: Supplementary file 1 — Additional file 1: Figure S1. A summary of the micro plasma processing system. [file 11671_2021_3478_MOESM1_ESM.pdf]
